# Sanghuangporus sanghuang extract extended the lifespan and healthspan of *Caenorhabditis elegans via* DAF-16/SIR-2.1

**DOI:** 10.3389/fphar.2023.1136897

**Published:** 2023-04-20

**Authors:** Zhenghan Dong, Yachao Wang, Cuiting Hao, Yuan Cheng, Xi Guo, Yanyu He, Yueyue Shi, Shuang Wang, Yunqi Li, Wei Shi

**Affiliations:** ^1^ Key Laboratory for Molecular Enzymology and Engineering, The Ministry of Education, Jilin University, Changchun, Jilin, China; ^2^ College of Life Sciences, Jilin University, Changchun, Jilin, China

**Keywords:** *Sanghuangporus sanghuang*, antiaging, *Caenorhabditis elegans*, antistress, daf-16, sir-2.1

## Abstract

*Sanghuangporus Sanghuang* is a fungus species. As a traditional Chinese medicine, it is known for antitumor, antioxidant and anti-inflammatory properties. However, the antiaging effect of *S. Sanghuang* has not been deeply studied. In this study, the effects of *S. Sanghuang* extract (SSE) supernatants on the changes of nematode indicators were investigated. The results showed that different concentrations of SSE prolonged the lifespans of nematodes and substantially increased these by 26.41%. In addition, accumulations of lipofuscin were also visibly reduced. The treatment using SSE also played a role in increasing stress resistance, decreasing ROS accumulations and obesity, and enhancing the physique. RT–PCR analysis showed that the SSE treatment upregulated the transcription of *daf-16*, *sir-2.1*, *daf-2*, *sod-3* and *hsp-16.2*, increased the expression of these genes in the insulin/IGF-1 signalling pathway and prolonged the lifespans of nematodes. This study reveals the new role of *S. Sanghuang* in promoting longevity and inhibiting stress and provides a theoretical basis for the application of *S. Sanghuang* in anti-ageing treatments.

## 1 Introduction

Ageing is an irreversible physiological process of organisms that is caused by increased incidences of various diseases and deterioration of physiological functions, which ultimately lead to death. According to the World Health Organization, the number of people suffering from age-related diseases is expected to nearly double in the next 20 years (de Cabo et al., 2014). Therefore, foods and drugs that can slow the ageing process are always an important research topic. Traditional Chinese medicine has long been thought to strengthen the body, and some studies have also shown that both Chinese medicine and its extracts play an important antiaging role (Liang and Yin, 2010). The consumption of traditional Chinese medicine as an adjunctive therapy against ageing has broad prospects.

*Caenorhabditis elegans* (*C. elegans*) nematodes with a full growth length of 1 mm are now extensively utilized as animal models in research on ageing and some neurodegenerative diseases, such as Parkinson’s and Alzheimer’s diseases (Alexander et al., 2014; Cooper and Van Raamsdonk, 2018; Shen et al., 2018). *C. elegans* possesses several traits: a short lifespan of approximately 2–3 weeks, large number of offspring, simple physiology, small size, invariant nervous system and fully sequenced genome (Park et al., 2017). Moreover, *C. elegans* are easy to culture in the laboratory and in experiments where *C. elegans* are free of ethical concerns (Papaevgeniou et al., 2017). Because of these advantages, *C. elegans* is widely used all over the world as an ideal model organism and its use has led to numerous breakthrough discoveries in the field of ageing research.

Currently, most of the physical characteristics of *C. elegans* have been discovered. The most suitable growth temperature for *C. elegans* is at 20°C, where it develops through four larval stages (e.g., L1–L4) from eggs to adults. This short process lasts approximately three days (Alexander et al., 2014). Adult *C. elegans* are mostly hermaphrodites; mature male adults account for only 0.05% of the population and contain constant numbers of 959 and 1,031 somatic cells, respectively (Kyriakakis et al., 2015). As the first animal whose genome has been completely sequenced, the *C. elegans* genome contains more than 65% of the genes associated with humans with many signalling pathways that have already been well studied (Shen et al., 2018). The insulin and IGF-1 signalling (IIS) pathway, which is highly conserved to modulate ageing and longevity, is the focus of research by scientists (Tissenbaum, 2018). The IIS pathway is a signal transduction cascade that contains the pivotal downstream Forkhead Box O transcription factor (DAF-16), an insulin/IGF-1 receptor (DAF-2), and phosphoinositide 3-kinase (AGE-1) (Sun et al., 2017). They are all important targets for ageing research.

*Sanghuangporus*, a group of highly prized edible and medicinal macrofungi belonging to Hymenochaetaceae, *Basidiomycota*, has been used to treat various diseases in China for nearly 2000 years (Zhou et al., 2015; Cai et al., 2019). *Shennong’s Herbal Classic of Materia Medica* and other ancient Chinese medical books recorded that long-term use of *Sanghuangporus* can clear away heat and toxic materials, improve digestion and prolong life (Cai et al., 2019). Due to its particular pharmacological effects and high level of safety (Huo et al., 2020), *Sanghuangporus* has attracted extensive attention from scholars worldwide in recent years (Jiang et al., 2021). Many studies have shown that *Sanghuangporus sanghuang* has excellent antitumor, anti-inflammatory, and antioxidant activities and hypoglycaemic effects (Lin et al., 2017a; Lin et al., 2017b; Cai et al., 2019; Wan et al., 2020); its potential as a treatment for diabetes and gout has also been revealed in recent years (Guo et al., 2021; Hou et al., 2021); polysaccharides, polyphenols, pyrones, and terpenes are the main metabolites that are responsible for these medicinal functions (Cai et al., 2019). In recent decades, from the first time that “*Sanghuang*” has been referred to by the scientific fungal genus name *Sanghuangporus*, the commercialization and industrialization of *S. sanghuang has* also developed rapidly (Zhou et al., 2020). Currently, wild *Sanghuang* has not only been extensively collected, but the scale of cultured *Sanghuang* production has also expanded. Recent studies have shown that cultured *Sanghuang* can be used as an alternative to wild *Sanghuang* used in nutraceutical medicine (Wen et al., 2019). “Sanghuang Tea,” “Sanghuang Wine,” “Sanghuang Candy” and additional highly processed *Sanghuang* products are enthusiastically welcomed by consumers and are even being considered as supplementary foods during chemo- and radio-therapies during cancer treatments in Korea (Han et al., 2016a). Nevertheless, the existing research on *Sanghuang* is still inadequate, and most previous reports have mainly focused on the cultivation and extraction of *S. Sanghuang*, while its effects on laboratory animals are less-well reported. To date, there are no reports regarding the use of *C. elegans* as an *in vivo* model to explore the physiological effects of *Sanghuangporus Sanghuang*.

## 2 Materials and methods

### 2.1 Preparation of SSE extracts

*S.Sanghuang* was cultured in a 5-litre fermentation tank (Baoxing Bioengineering Equipment Co., Ltd., Shanghai, China) according to the methods used in our laboratory (Guo et al., 2021). One week later, the fermentation liquid was centrifuged to obtain supernatant and mycelial fractions. The mycelia were completely dehydrated in a 60°C oven, and the products were then boiled in water for 2 h. To obtain aqueous extracts, the mixture was centrifuged at 12,000 rpm for 15 min, and the supernatant was separated. The aqueous extracts were freeze-dried with a vacuum freeze-dryer to obtain the dry powder of *SSE*. The dry powder was stored in a 4°C refrigerator, and solutions with different concentrations were prepared as required during the experiment.

### 2.2 Preparation of NGM solid medium and OP50

The NGM medium solution was prepared according to the literature (Hou et al., 2019) and then placed into an autoclave for sterilization. It was then poured quickly onto plates and allowed to cool until solidification. The plates were inverted and placed in a 4°C refrigerator until use. OP50 is an uracil trophic deficiency type *E. coli*, which prevents *Escherichia coli* from obstructing the growth of nematodes (MacNeil et al., 2013). The OP50 bacteria were streaked onto solid LB medium and then cultured in a 37°C incubator for 24 h. A single colony was selected and placed into 100 mL of LB liquid medium and cultured in a 120 rpm/min 37°C incubator for 8–10 h until the OD600 was approximately 1.5. The bacteria were then stored in a 4°C refrigerator until use.

### 2.3 Culture and synchronization of *Caenorhabditis elegans*

All strains were obtained from the *Caenorhabditis* Genetics Center (CGC). Wild-type N2 *C. elegans* were cultured on NGM media covered with *E. coli* OP50 as food and incubated in a 20°C incubator. The nematode lysis buffer contained 5% NaClO and 5 M NaOH, and the nematodes that were collected from the M9 buffer were mixed with the lysis buffer. The eggs were centrifuged at 300 rpm/min and cultured on new NGM media without OP50. Two days later, all nematodes were in the Dauer stage (Wong and Roy, 2020) and were then transferred to plates covered with OP50 to obtain age-synchronized nematodes.

### 2.4 Effects of SSE on the growth of the OP50 strain

OP50 monoclones were placed into 100 mL of LB liquid medium at 37°C and incubated for 12 h. In addition, three bottles of LB liquid medium were prepared, and one of the bottles was used as a blank control. A 100 μL volume of was added OP50 to each of the remaining 2 bottles and an additional 100 µL of *SSE* was added to one bottle. The cells were cultured in an incubator at 180 rpm/min and 37°C for 8 h, and the OD600 values were measured hourly.

### 2.5 The pharyngeal pumping assay of *Caenorhabditis elegans*

The NGM media of *S. The Sanghuang* treatment group were covered with 150 μL of *SSE* solutions with different concentrations and 150 μL of OP50; meanwhile, the control group was covered with 150 μL of M9 buffer solution and 150 μL of OP50. Five-day-old nematodes were placed on media with different concentrations of *SSE* solution (e.g., 50, 25 and 10 mg/mL), and the pharyngeal pumping rates were observed under a microscope.

### 2.6 Lifespan assay

Growing synchronized nematodes at the L4 stage were picked from the NGM plates, with 50 nematodes per plate, and at least 3 plates were used for both the control and treatment groups. All of the plates were covered with 150 μL of OP50 and *S. angusthuang* solutions with different concentrations (e.g., 50, 25 and 10 mg/mL), while the control group used M9 buffer instead of medicine. Then, 50 μM 5-fluoro-20-deoxyuridine (FUDR) was added to each plate to prevent nematode reproduction. The time at which the worms were first treated with *SSE* was recorded as day 0, and the plates were then placed in a 20°C incubator. The nematodes were transferred to new treatment plates every day to eliminate interference from offspring nematodes. If a needle touched a nematode head several times and there was no response, this nematode was recorded as dead. The number of dead nematodes was recorded until all of the nematodes on each plate were dead, and the survival rates of *C. elegans* were calculated. The worms that died in the process of selection, became desiccated, were stuck to the sides of plates or experienced other abnormal deaths were excluded from the statistics. GraphPad software was used to plot the survival rates, and the data are reported as the mean ± SD. The experiments were independently repeated at least three times.

### 2.7 Lipofuscin assay

Lipofuscin is a marker of senescence in nematodes that can be analysed by using fluorescence microscopy (Kampkotter et al., 2007; Shen et al., 2018). The synchronized L4-stage nematodes were cultured on NGM plates that were covered with *SSE* solutions of various concentrations. After 10 days of treatment, the worms were first anaesthetized with 100 μL of levamisole (1 M), and the fluorescence intensities of lipofuscin were then observed under a fluorescence microscope and recorded. ImageJ software was used to analyse the relative fluorescence intensities.

### 2.8 Athletic ability assay

During the lifespan assay, we observed the athletic abilities of nematodes on each plate on the 8th, 12th, 16th and 20th days of adulthood. *C. elegans* individuals were given a mechanical stimulus with a platinum wire and according to the response, it was possible to judge their athletic abilities. “Normal” nematodes moved rapidly without being touched by the wire, “sluggish” nematodes moved only after their heads were touched, and “Immobile” nematodes, even when their heads were touched, moved only slightly. The numbers of nematodes that were in these three states were recorded, and their athletic abilities were calculated.

### 2.9 Reproduction assay

Synchronized L4-stage nematodes were picked from the control and treatment groups, with only one worm per plate. The worms were cultured under normal conditions, and the time of the first treatment with *SSE* was recorded as the first day. The worms were transferred to new plates every day and were observed under a light microscope, and the numbers of eggs were calculated. When all of the worms stopped laying eggs, the number of offspring from each worm was counted and summed to obtain the total number of offspring and the reproduction rate was calculated.

### 2.10 Body length and movement speed assay

Synchronized L4-stage nematodes were picked from the control and treatment groups, with at least 100 nematodes per plate. Ten worms were randomly selected from each plate every other day for microscopic observations, and this procedure was repeated three times to decrease the error levels. We obtained pictures and videos, and ImageJ software was used to determine the body lengths and calculate the number of sinusoidal movements in 30-s intervals.

### 2.11 Lipid metabolism assay

The transparent bodies of *C. elegans* enable visualization of fat stores by using dye-labelled imaging (Shen et al., 2018). Here, we used Oil Red O to dye the worm fat. We also cultured synchronized L4-stage *C. elegans* from the control and treatment groups for 5 days and then collected these nematodes into 1.5-mL tubes. They were then starved for 6 h to burn off excess fat. Paraformaldehyde was used to immobilize the nematodes, which were cleaned with isopropyl alcohol after repeated freeze-thaw cycles. The nematodes were immersed in Oil Red O for 12 h to complete the dying process. The nematodes were then repeatedly rinsed and photographed under a microscope. The fat metabolism speeds were analysed with ImageJ software.

### 2.12 Thermotolerance assay

We cultured synchronized L4-stage *C. elegans* from the control and treatment groups for 5 days. They were transferred to new NGM plates and placed in a 37°C incubator to cause exposure to thermal stress. Every 1.5 h, nematode deaths were observed, and nematodes were determined to dead if they did not respond to repeated stimulation. When all worms were dead, the survival rates under thermal stress were calculated. Each plate contained at least 40 worms. These experiments were repeated at least three times.

### 2.13 Inoxidizability assay

The preliminary treatment was the same as for the thermotolerance assay. Then, the worms were transferred to plates that were covered with 100 μL of 3% H2O2. Every 2 h, nematode deaths were observed, and nematodes were determined to be dead if they did not respond to repeated stimulation. We recorded the numbers of dead nematodes and calculated the survival rates. Each plate contained at least 30 worms. These experiments were repeated at least three times.

### 2.14 Intracellular ROS levels

According to a previous report (Back et al., 2012), we quantitatively analysed the ROS levels in nematodes with a specific fluorescent molecular probe (DCFH-DA) that used a Reactive Oxygen Species Assay Kit (Beyotime Biotechnology, Shanghai, China). We also cultured synchronized L4-stage *C. elegans* in the control and treatment groups for 5 days and then collected these nematodes in 1.5-mL centrifuge tubes with M9 buffer. The nematodes were treated according to the ROS Assay kit instructions, and photographs were taken under a fluorescence microscope. ImageJ software was used to analyse the relative fluorescence intensities.

### 2.15 Real-time RT–PCR

We cultured synchronized L4-stage *C. elegans* from the control and treatment groups for 7 days, with approximately 500 worms per plate. The nematodes were ground in a mortar filled with liquid nitrogen by using TRIzol reagent to extract total RNA. The RNA qualities and concentrations were determined with a nucleic acid analyser. First-Strand cDNA Synthesis SuperMix (TransGen Biotech, Beijing, China) was used to obtain cDNA, and the PCR conditions were 42°C for 15 min and then 85°C for 5 s. An Applied Biosystems 7,500 Real-Time PCR System (Applied Biosystems, Waltham, United States) was used to conduct quantitative real-time PCR with SYBR Green Supermix (Takara Biotechnology, Dalian, China). The 2^−ΔΔCT^ method was used to analyse the gene expression levels.

### 2.16 Metabonomics

Wild-type *C. elegans* treated with 25 mg/mL SSE for seven days were assessed by the APExBIO Company (Shanghai, China). Each sample was about 50mg, over two thousand nematodes, and each group had at least three samples. After pre-processing, the samples were analyzed for inter-group differentiation.

### 2.17 Statistical analysis

All of the data are presented as the mean values ± SDs that were obtained from at least three replicates. GraphPad Prism 8.02 (GraphPad Software, CA, United States) was used to examine the statistical significance of the differences and to construct the graphics presented. T tests were used to perform the significance tests, and *p ≤ 0.05, **p ≤ 0.01, ***p ≤ 0.001 were considered to be statistically significant.

## 3 Results

### 3.1 SSE prolonged the lifespan of *Caenorhabditis elegans*

**FIGURE 1:**
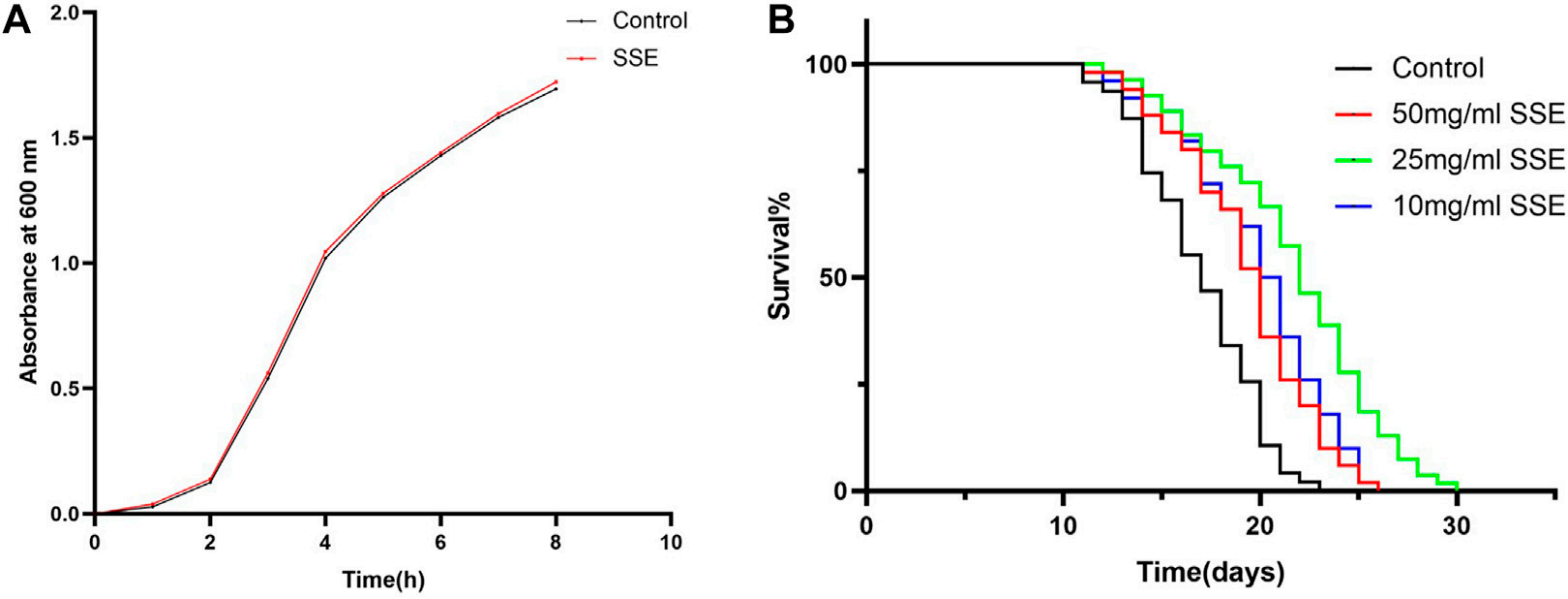
Effect of SSE on the lifespan of N2 wild-type *Caenorhabditis elegans*. **(A)** Effect of *SSE* on the propagation of E. coli OP50. **(B)** The time that the worms were first treated with S. Sanghuang was recorded as day 0; SSE treatments noticeably prolonged the survival times of nematode (*p* < 0.05). The experiment was repeated at least three times.

Food is one of the most important environmental factors, and dietary restrictions have a great influence on the ageing and other physiological activities of nematodes (Kenyon, 2010; Zhang et al., 2021). One potential concern is that *SSE* might have limited feeding by affecting the growth of *E. coli*, so we first verified whether *SSE* had an effect on *E. coli* OP50 inhibition. We found that the growth rate of the OD600 value of the *SSE* groups nearly overlapped with that of the control group (Figure 1A), which indicated that *SSE* does not interfere with the regular growth of *E. coli* OP50.

Lifespans provide a key piece of evidence for the ageing of nematodes, so we cultured wild-type *C. elegans* N2 with different SSE concentrations (e.g., 50, 25, and 10 mg/mL) to investigate whether SSE affected the lifespan of *C. elegans*. As shown in Figure 1B and Table 1, the survival curves of all nematodes were nearly the same until the 13th day, but after day 14, the survival curves for the SSE treatment groups shifted to the right compared to the control group, and these shifts were especially obvious for the 25 mg/mL group. The mean lifespan for the control group was 16.23 ± 0.36 days, while the 25 mg/mL group survived the longest, reaching 20.52 ± 0.34 days, which was an increase of 26.41%. The lifespans of the 50 and 10 mg/mL groups also increased by 12.28% and 16.27%, respectively. There was also a visible increase in the maximum lifespan. As indicated above, we hypothesized that SSE treatments effectively prolonged the lifespan of nematodes, and other indicators were selected to further explore the influence of SSE on ageing.

**TABLE 1:** Effects of *SSE* treatments on the lifespans of *Caenorhabditis elegans* (mean ± SD, *n* = 3).

Group	Mean Lifespan(d)	% of control	Max lifespan (d)
Control	16.23 ± 0.36	100 ± 1.97	21.3 ± 0.58
50 mg/mL SSE	18.23 ± 0.10	112.28 ± 0.63	24.7 ± 1.15
25 mg/mL SSE	20.52 ± 0.34	126.41 ± 2.10	28.7 ± 0.58
10 mg/mL SSE	18.85 ± 0.05	116.27 ± 0.16	25.0 ± 1.00

### 3.2 SSE had no effect on the reproductive capacity of *Caenorhabditis elegans*

**FIGURE 2:**
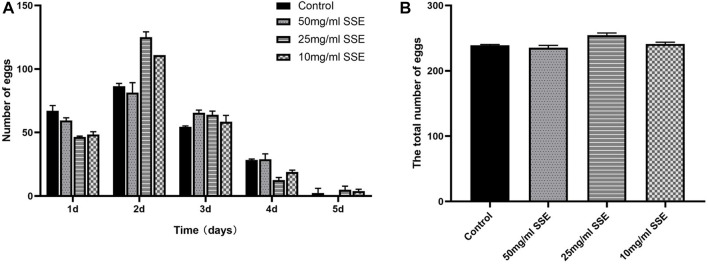
Effects of SSE on the reproductive capacity of *Caenorhabditis elegans*. **(A)** Numbers of eggs laid per day after *SSE* treatment. **(B)** The SSE treatment had no obvious effect on reproduction (*p* > 0.05). All data were determined in triplicate (mean ± SD, *n* = 3).

Reproduction is one of the most important tasks for living beings; the reproductive capacity is therefore an important indicator of health and ageing. Nematodes generally have higher reproductive capacities in the first 3 days of the L4 stage, and with increasing age, these capacities continuously decrease and stop at approximately the end of the fifth day (Figure 2A). As shown in Figure 2B, the average spawning production of the control group was 239.5 ± 1.4, and there were no significant differences in the total number of eggs produced among the groups that were treated with different *SSE* concentrations, namely, 215.5 ± 2.9, 254.5 ± 2.6, and 241 ± 3.1 in the 50, 25, and 10 mg/mL groups, respectively. The results showed that the *SSE* treatments had no effect on nematode reproduction.

### 3.3 SSE decreased lipofuscin accumulation in *Caenorhabditis elegans*

**FIGURE 3:**
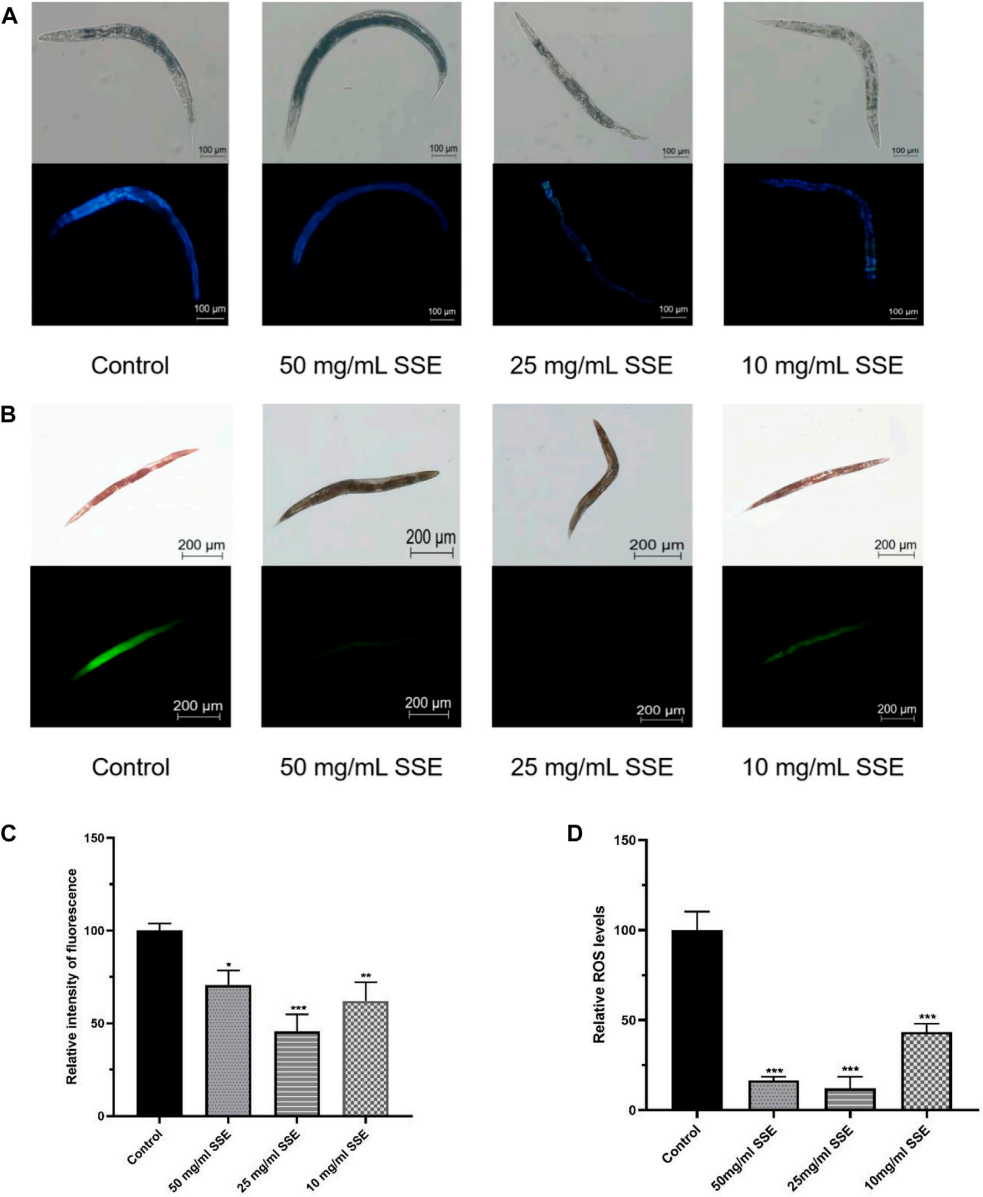
Effects of *SSE* on lipofuscin and ROS accumulation. **(A)** The lipofuscin accumulations were measured with a fluorescence microscope **(B)** Representative pictures of the ROS accumulations of *Caenorhabditis elegans* **(C)** ImageJ software was used to determine the relative fluorescence intensities. **(D)** ImageJ software was used to determine the relative fluorescence intensities. *p ≤ 0.05, **p ≤ 0.01, ***p ≤ 0.001.

Lipofuscin accumulations are an important sign of nematode senescence; the senescence degrees of nematodes can be determined by the fluorescence intensities of lipofuscin determined under a microscope. We found that the nematodes treated with *SSE* exhibited lighter colours under the microscope, which indicated that the *SSE* treatments reduced lipofuscin accumulations (Figure 3A). Compared with the control group, the fluorescence intensities decreased by 29.2%, 54.5% and 37.9% at *SSE* treatment concentrations of 50, 25, and 10 mg/mL, respectively (Figure 3C).

### 3.4 SSE decreased ROS accumulation in *Caenorhabditis elegans*

ROS accumulations are considered to be one of the important causes of nematode senescence, and decreasing ROS production can effectively delay ageing. Dichlorofluorescin-diacetate (DCFH-DA) was used to determine the ROS levels in nematodes. Intracellular ROS can oxidize non-fluorescent DCFH into fluorescent DCF; thus, the ROS levels in nematodes can be determined from the fluorescence intensities. As shown in Figure 3B, the green fluorescence of nematodes that were treated with *SSE* was substantially dimmer than that of the control group. By using the software to process the images, the observed ROS levels were reduced by 83.4%, 87.8% and 56.6% among the *SSE* groups treated with concentrations of 50, 25, and 10 mg/mL, respectively, compared to the control nematodes (Figure 3D). The results indicated that treatment with *SSE* effectively reduced ROS accumulations in nematodes.

### 3.5 SSE partly improved healthspan in *Caenorhabditis elegans*

**FIGURE 4:**
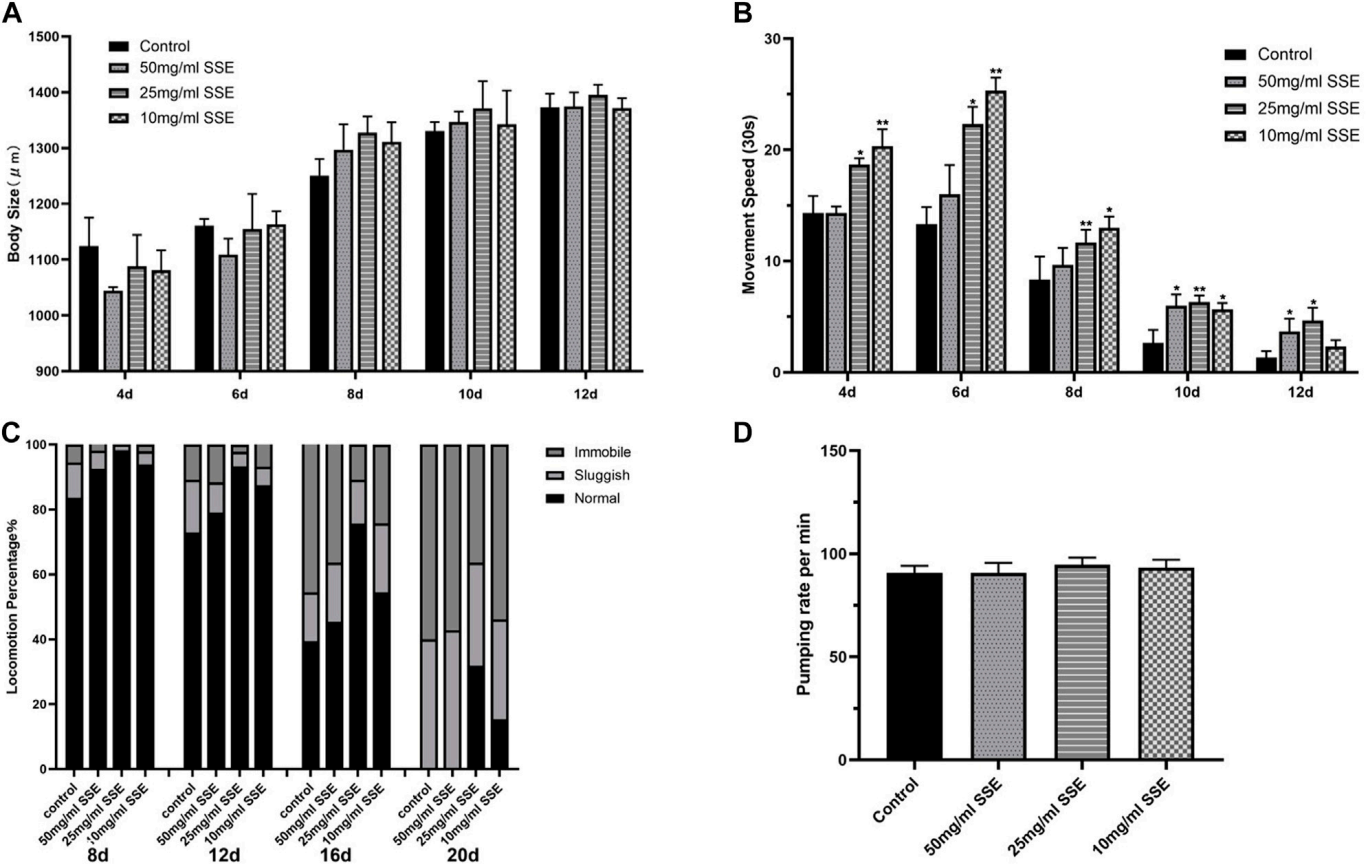
Effects of SSE on the Physiological Responses of *Caenorhabditis elegans*. Measurements of body size **(A)** and movement speed **(B)** from day 4 to day 12. **(C)** Effect of SSE on the motility of *Caenorhabditis elegans*. Motility was classified into three groups: Normal, worms exhibit spontaneous sinusoidal motions; Sluggish, worms move after touched; Immobile, hardly any movement. **(D)** Effect of SSE on pharyngeal pumping rate in N2 worms. *p ≤ 0.05, **p ≤ 0.01.

Body size, movement behaviour, and movement speed are all important indicators of the physiological responses of nematodes; their states are also closely linked to ageing and health. We first evaluated the body sizes of nematodes that were treated with *SSE*. When the nematodes were just beginning to develop (e.g., on days 4 and 6), the body sizes for the *SSE* treatment groups were shorter than those of the control group (Figure 4A). However, when the nematodes developed further (e.g., after day 8), the body sizes for the nematodes in the *SSE* treatment groups was visibly longer than those in the control group. On day 12, the vast majority of nematodes were fully developed, and their body sizes also tended to be stable. The results showed that there were no significant differences in body size among the groups; indeed, at puberty, the *SSE* treatments even promoted nematode growth.

In addition to measuring body lengths, we also measured the movement speeds of the worms. Obviously, the SSE-treated nematodes exhibited greater speeds than the control nematodes in both the infancy and mature periods. It is worth noting that the 10 mg/mL treatment was associated with greater increases in the early stage, but when the nematodes matured, the 25 and 50 mg/mL *SSE* groups exhibited more significant effects (Figure 4B). This may be related to the adaptation of nematodes to the *SSE* treatments. Meanwhile, we observed the motility characteristics of nematodes at different life stages (e.g., on days 8, 12, 16, and 20). All of the worms moved normally in the early stage of the life process, but as the worms aged, the proportions that could move freely decreased towards the end of life. The control group contained almost no functioning nematodes (Figure 4C). However, *SSE* treatments improved this situation. The worms in the control groups that still exhibited normal movement represented less than 50% of the initial number (on day 16), 70% of the worms treated with 25 mg/mL of *SSE* moved regularly, and the 50 mg/mL and 10 mg/mL groups also showed varying degrees of improvement. By day 20, the SSE-treated worms still exhibited better movement capabilities than those in the control group; 32% and 15% of the nematodes treated with 25 and 10 mg/mL, respectively, of *SSE* still moved spontaneously. Last, we compared the pharyngeal pumping rate of worms, either in the presence or absence of *SSE*. The result indicated that *SSE* did not affect the pharyngeal pumping rate of the worm (Figure 4D).

### 3.6 SSE accelerated lipid metabolism in *Caenorhabditis elegans*

**FIGURE 5:**
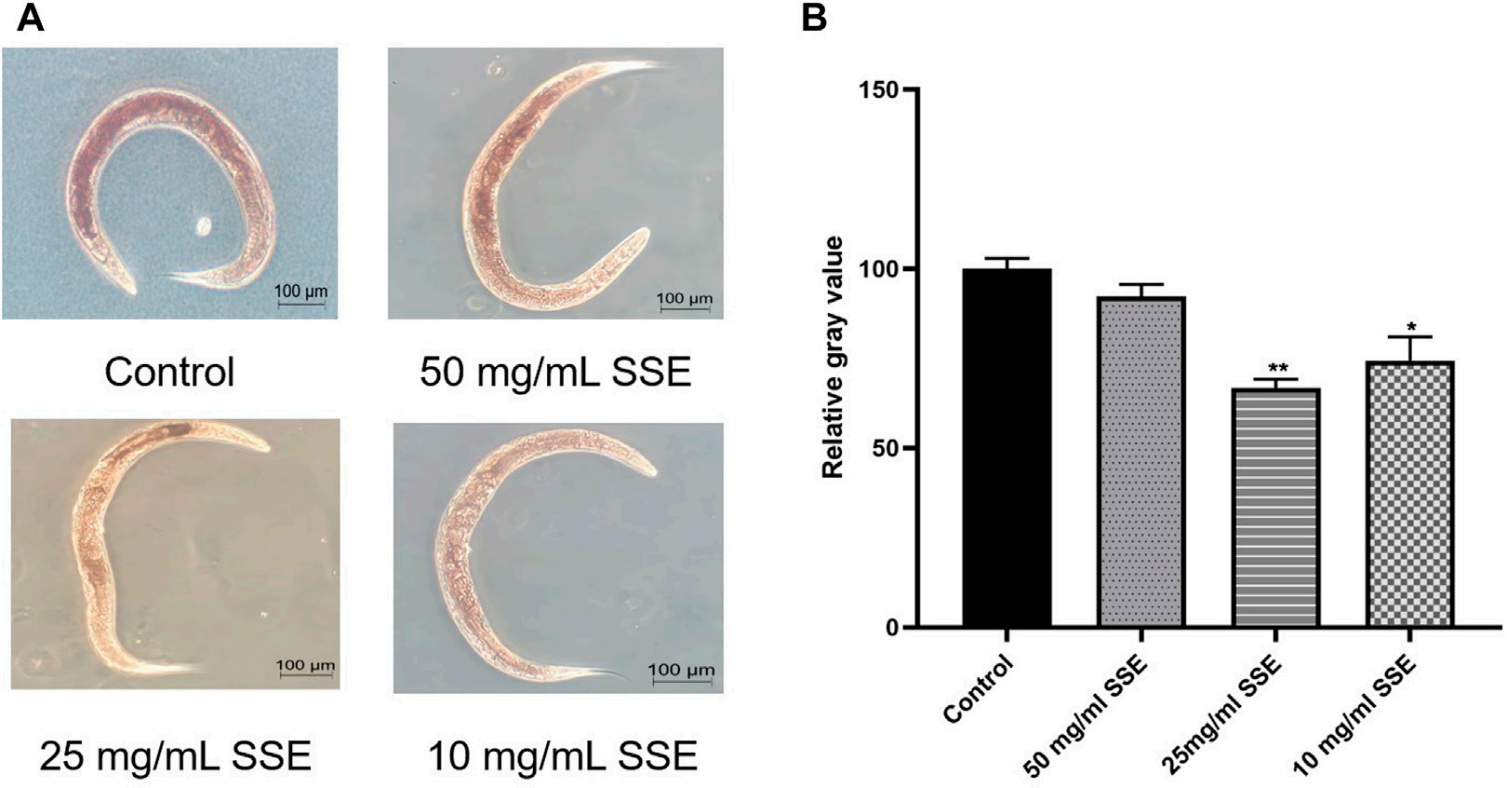
*SSE* accelerated the lipid metabolism of *Caenorhabditis elegans*. **(A)** Representative pictures of the results of nematode Oil red O staining. **(B)** ImageJ software was used to determine the relative grey values. *p ≤ 0.05, **p ≤ 0.01.

Obesity is very detrimental to health, and ageing also leads to fat accumulation, so accelerating the lipid metabolism provides significant health and antiaging benefits. Oil red O is a specific dye for lipid particles, and the brightness of the red colour observed under the microscope indicates the accumulated lipid amounts. As shown in Figure 5A, the control nematodes were deep red, whereas the red colours of nematodes that were treated with *SSE* became lighter, especially in the 25 mg/mL group, and the nematode bodies were nearly transparent. The average grey value analysis also proved that the *SSE* groups exhibited brighter red colours than the control group (Figure 5B). The results showed that *SSE* treatments can decrease lipid contents and accelerate lipid metabolism in *C. elegans*.

### 3.7 SSE-treated *Caenorhabditis elegans* increased their resistance to heat shock and oxidation

**FIGURE 6:**
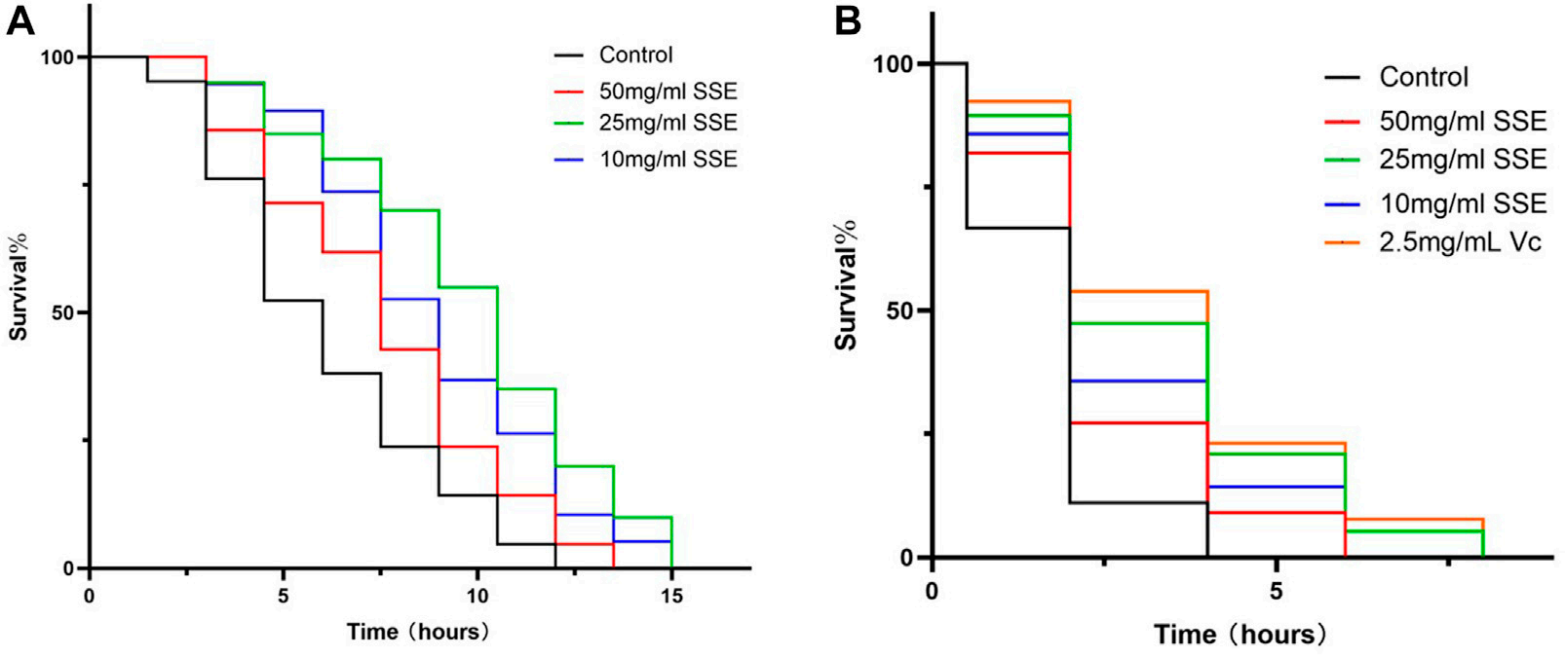
Effects of *SSE* on nematode stress resistance. **(A)** Survival times of nematodes when subjected to 37°C heat shock. **(B)** Survival times of nematodes under 3% H_2_O_2_, 2.5 mg/mL Vitamin C was used as a positive control.

Improved stress resistance in *C. elegans* is considered to be one of the most important manifestations of life extension. We first measured the survival times of nematodes at 37°C to investigate the effect of *SSE* treatments on heat shock resistance. As shown in Figure 6A and Table 2, the mean lifespan of the control nematodes was 4.52 ± 0.09 h (maximum of 10 ± 0.70 h), which was clearly lower than those in the *SSE* treatment groups. The group that survived longest was the 25 mg/mL group, in which the mean lifespan reached 7.97 ± 0.20 h (maximum of 13.5 ± 0 h), an increase of 76.19%. The *SSE* treatments at concentrations of 50 and 10 mg/mL also had clear effects, and the mean lifespans increased by 34.78% and 49.83%, respectively. These results revealed that *SSE* treatments could evidently decrease heat shock damage and increase the survival rates of *C. elegans*.

**TABLE 2:** Effects of *SSE* on the heat shock responses of *Caenorhabditis elegans* (mean ± SD, *n* = 3).

Group	Mean Lifespan(h)	% of control	Max lifespan (h)
Control	4.52 ± 0.09	100 ± 1.97	10 ± 0.70
50 mg/mL SSE	6.09 ± 0.30	134.78 ± 6.66	11.5 ± 0.70
25 mg/mL SSE	7.97 ± 0.20	176.19 ± 4.45	13.5 ± 0
10 mg/mL SSE	6.78 ± 0.22	149.83 ± 4.76	12.5 ± 0.70

Subsequently, we investigated whether *SSE* could improve the antioxidant capacity of *C. elegans*, and vitamin C was added as a positive control. The survival rates were similar to those obtained from the heat shock assay (Figure 6B; Table 3): the mean lifespan of the control nematodes was only 0.57 ± 0.05 h (maximum of 3.00 ± 1.00 h) in 100 μL of 3% H_2_O_2_. The 25 mg/mL group exhibited the best effect, with a mean lifespan of 1.64 ± 0.59 h (maximum of 5.67 ± 0.94 h), which represented an increase of 185.32%, and this was very close to that of the positive control group. The 50 and 10 mg/mL groups exhibited increases of 87.09% and 140.45%, respectively. *SSE* noticeably enhanced the antioxidant capacity of nematodes. Moreover, these results indicated that *SSE* can improve the survival ability of nematodes in adverse environments.

**TABLE 3:** Effects of SSE on the oxidant treatment responses of *Caenorhabditis elegans* (mean ± SD, *n* = 3).

Group	Mean Lifespan(h)	% of control	Max lifespan (h)
Control	0.57 ± 0.05	100 ± 9.15	3.00 ± 1.00
50 mg/mL SSE	1.07 ± 0.08	187.09 ± 13.82	5.00 ± 1.00
25 mg/mL SSE	1.64 ± 0.59	285.32 ± 10.21	5.67 ± 0.94
10 mg/mL SSE	1.38 ± 0.09	240.45 ± 16.09	5.00 ± 1.00
2.5 mg/mL Vc	1.84 ± 0.08	320.19 ± 13.31	7.00 ± 1.00

### 3.8 SSE treatment influenced mRNA expression in *Caenorhabditis elegans*

**FIGURE 7:**
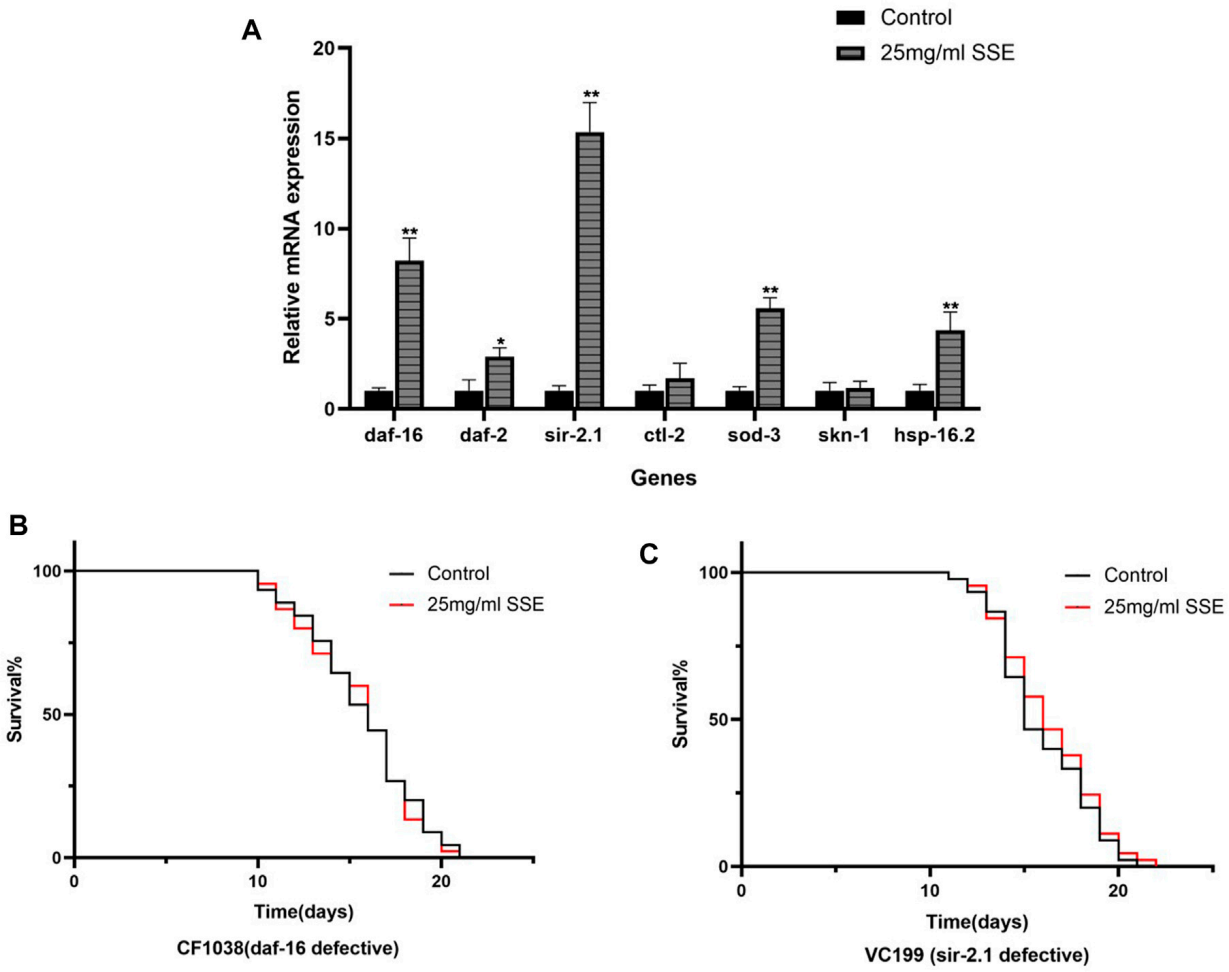
Mechanism of SSE-mediated longevity. **(A)** Effect of orange extracts on age-related gene expressions in *Caenorhabditis elegans*. **(B)** Effect of SSE treatment on lifespan of daf-16 defective (CE1038) worms. **(C)** Effect of SSE treatment on lifespan of sir-2.1 defective (VC199) worms. *p ≤ 0.05, **p ≤ 0.01.

The above results indicated that *SSE* can effectively retard ageing in nematodes, and we further explored the possible mechanisms at the transcriptional level. We selected several genes that are associated with ageing and determined their expressions, including daf-16 (Fork-head domain-containing protein), daf-2 (Insulin-like receptor subunit beta), sod-3 (Superoxide dismutase [Mn] 2), ctl-1 (Peroxisomal catalase 1), sir-2.1 (Deacetylase sirtuin-type domain-containing protein), skn-1 (BZIP domain-containing protein), and hsp-16.2 (Heat shock protein hsp-16.2) by using real-time PCR. These results showed that the SSE treatments improved the expressions of daf-16, daf-2, sir-2.1, sod-3 and hsp-16.2 but did not have significant effects on the ctl-1 and skn-1 expressions (Figure 7A).

### 3.9 Daf-16 and Sir-2.1 was required for SSE-Mediated lifespan extension

The forkhead transcription factor, DAF-16, a downstream target of the insulin/IGF-I signaling pathway in *C. elegans*, is indispensable both for lifespan regulation and stress resistance (Li et al., 2007). Sir-2.1 is critical regulator of FOXO-mediated transcription in response to oxidative stress (Hamilton et al., 2005). RT-PCR results show that the expressions of daf-16 and sir-2.1 increase significantly, so we speculate that these two genes are the key factors in SSE extending the lifespan of *C. elegans*. We treated *daf-16*(CF1038) and *sir-2.1*(VC199) mutant worms with 25 mg/mL SSE. Results shown that the SSE-mediated lifespan extension disappeared, suggesting SSE prolonged the lifespan of nematodes in daf-16 and sir-2.1 dependent manner (Figures 7B, C).

### 3.10 Metabolic changes of SSE treated N2 C.elegans

**FIGURE 8:**
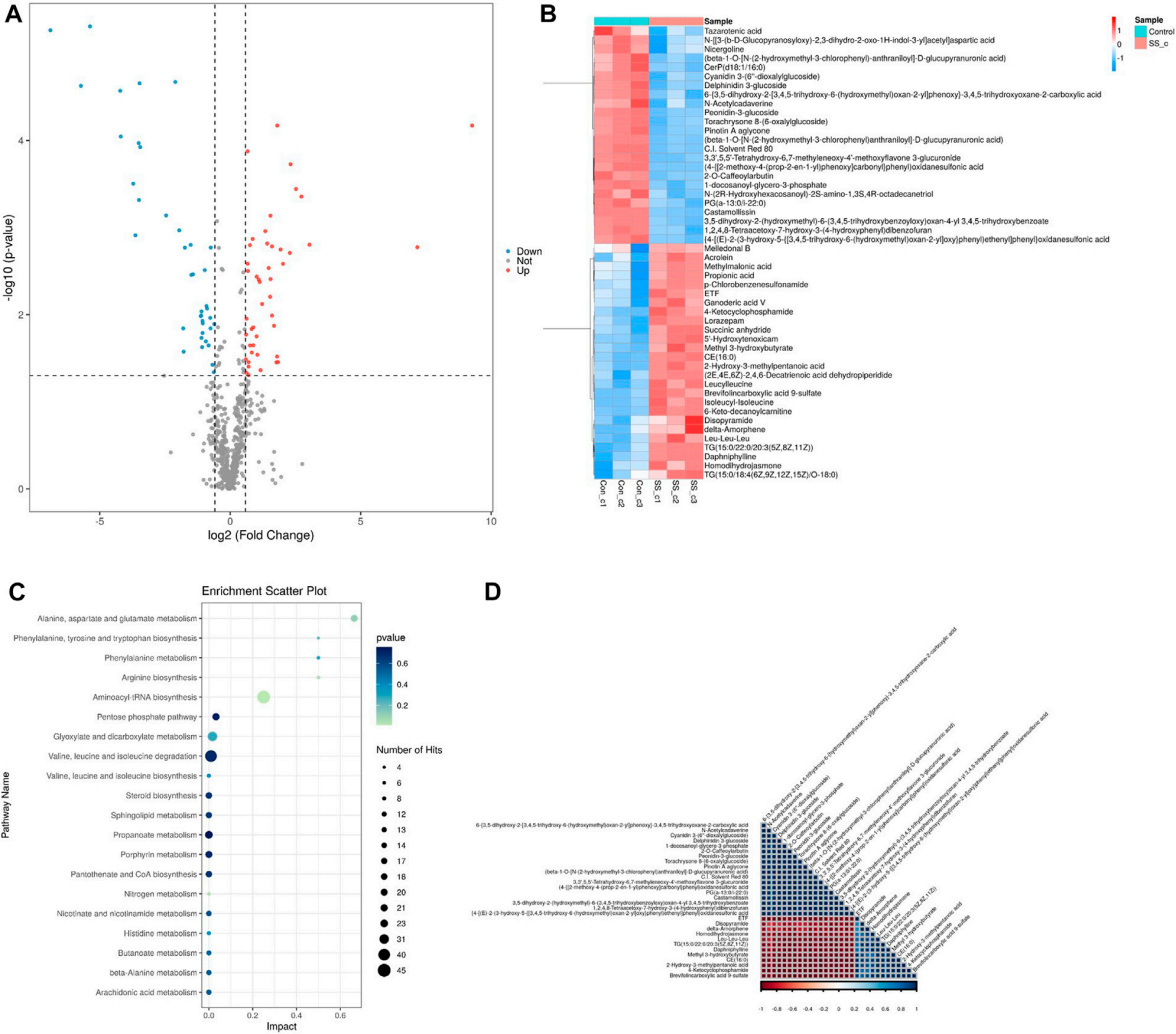
Metabolomics analysis on nematodes treated withSSE for 7 days. **(A)** 49 metabolites were upregulated and 36 metabolites were downregulated following the SSE treatment (*p*-value < 0.05 and Fold Change ≥2). **(B)** Hierarchical clustering heat map of differential metabolites **(C)** The KEGG analysis of differentially expressed metabolites in SSE-treated *Caenorhabditis elegans*. **(D)** Pearson Correlation coefficient is used to quantify the linear correlation between all differential metabolites. The closer the square colour of the two metabolites is to red, the stronger the positive correlation between the two metabolites is; conversely, the closer the square colour is to blue, the stronger the negative corelation between the two metabolites is.

We carried out metabonomics to understand the mechanism of SSE-mediated lifespan extension. Following 7 days of SSE treatment, 49 metabolites were differentially upregulated, and 36 metabolites were downregulated compared to control group (*p*-value < 0.05 and Fold Change ≥2). Amino acid metabolites, including leucine and isoleucine, were upregulated (Figures 8A, B). Leucine can activate Sirt1 by lowering its KM for NAD^(+)^, the homolog of mammalian SIRT1 in *C. elegans* is *sir2.1*, so SSE may extend the lifespan of *C. elegans* by activating *sir-2.1* through the increase of leucine metabolism (Raynes et al., 2012; Banerjee et al., 2016).

The KEGG (Kyoto Encyclopedia of Genes and Genomes) analysis revealed dramatic changes in the metabolism of amino acid (Figure 8C). It has been reported that the increase of amino acids can prolong the lifespan of nematodes and the extension is dependent on *daf-16*. SSE increases the rate of amino acid metabolism in *C. elegans*, which may also be a factor in lifespan extension. Finally, we quantified the linear correlation between the various metabolites (Figure 8D).

## 4 Discussion

*S. sanghuang* is a widely-used traditional Chinese medicine with an ancient history, and its good antitumor effect has been widely recognized by the international community in recent years (Han et al., 2016b; Guo et al., 2022). Its antioxidant and anti-inflammatory activities were gradually discovered (Lin et al., 2017a); previous studies in our laboratory have also shown that it has a significant therapeutic effect on gout (Guo et al., 2021). The polyphenolic compounds, Sanghuang triterpenoids and Phellinus linteus polysaccharides have all been proven to have good pharmacological effects (Suabjakyong et al., 2015; Lin et al., 2017b; Cai et al., 2019); however, the specific therapeutic mechanism of *S. Sanghuang* is not completely clear. To facilitate quantitative analyses, we created a dry powder from *S. Sanghuang* and prepared solutions with different concentrations. In the first experiment, we discovered that concentrations above 100 mg/mL had certain negative influences on nematode growth, which may be because *SSE*, as a medicinal material, is slightly toxic at high concentrations. Therefore, we selected concentrations of 50, 25 and 10 mg/mL for use in the experiments.

Many studies have confirmed that dietary restrictions prolong the lifespan of *C. elegans* (Hansen et al., 2008). If *SSE* treatment affected the diet of nematodes, it was not possible to prove that *SSE* itself altered the physiological functions of nematodes. Therefore, we first explored the effects of the *SSE* treatments on *E. coli* growth. The reproduction of *E. coli* was not disturbed by *SSE*, and the feeding frequencies of the nematode pharyngeal pumps also indicated that the nematodes ate as usual. These results rule out the possibility of dietary influences on nematode development and improve the authenticity of our subsequent experimental results.

Next, we tested the effect of *SSE* on nematode lifespans under normal conditions. The average lifespan of nematodes in the control group was 16.23 ± 0.36 days, and the maximum lifespan was 22 days. This was consistent with the average lifespan of *C. elegans* (approximately 2–3 weeks) reported in the literature (Son et al., 2019). However, the survival curves of nematodes in all of the *S. Shuang* treatment groups shifted substantially to the right, especially the 25 mg/mL group. This result provides the most direct evidence for the antiaging effects of *SSE*. Then, we measured the accumulations of lipofuscin, an age pigment that is produced as ageing progresses in nematodes (Papaevgeniou et al., 2017). Compared with the bright blue colour observed for the control group, nematodes that were treated with *SSE* exhibited only faint fluorescence, which indicated a significant reduction in lipofuscin accumulations. Studies have shown that antioxidant activities may decrease lipofuscin accumulations (Kampkotter et al., 2007), and we speculated that this might also be the mechanism used by *SSE* to decrease lipofuscin accumulations.

Evolutionary biology suggests that reproduction exacts a cost of physical maintenance, which reduces longevity (Mukhopadhyay and Tissenbaum, 2007). Some studies have also shown that using drugs that cause nematodes to be infertile can prolong their lifespans (Wan et al., 2017). From the perspective of energy metabolism, when nematodes do not need to expend energy for reproduction, they may have more energy to fight ageing. However, no reductions in fertility were observed in our experiments, and the total numbers of eggs laid by the nematodes in the control group and *SSE* treatment groups were generally the same. This phenomenon may be because *SSE* supplements the energy cost of reproduction or reduces the effect of reproduction on longevity.

A series of physiological functions, including body size, movement behaviour, and movement speed, are closely related to ageing (Wang et al., 2020). The results showed that treatments with *SSE* did not harm nematode development; instead, their development was even slightly promoted. We simultaneously measured the movement speeds of different nematodes and found that the nematodes in *SSE* treatment groups exhibited faster movement speeds in each period, which may be because the *SSE* treatments provided the worms with stronger bodies. The experiment on mobile behaviours exhibited similar results, and SSE-treated nematodes were more vigorous and youthful at different stages of their life cycles. At the last stage of the worms’ lives (e.g., at 20 days), some nematodes in the 25 and 10 mg/mL groups could still move as “Normal”, whereas the motility of the control worms was only scored as “Sluggish” or “Immobile”. All of these results indicate that *SSE* can improve the body functions of nematodes, and stronger bodies might be the key to fighting ageing.

The ability of animals to respond to changing environments is called stress tolerance, and researchers have shown that the resistance to multiple types of stress peaks during early adulthood and then declines with age (Dues et al., 2016). We exposed the nematodes separately to 37°C heat shock and 3% H2O2 oxidizability conditions. The survival rates of nematodes in *SSE* groups greatly improved, both for heat shock and oxidation. The results showed that the *SSE* treatments improved the stress resistance and survival ability of nematodes in harsh environments. Dylan J. Dues et al. (2016) found that *daf-16* was needed to maintain induced thermotolerance with age, which is also consistent with our subsequent experimental results. Obesity and ageing are two sides of the same coin and share a similar spectrum of phenotypes, such as compromised genomic integrity, impaired mitochondrial function, accumulation of intracellular macromolecules, weakened immunity, shifts in tissue and body composition, and enhanced systemic inflammation (Tam et al., 2020). This means that reducing obesity also slows down ageing. Oil red O staining showed that the *SSE* treatments could reduce fat accumulations in nematodes, and a decline in obesity may be able to boost the antiaging effect of *SSE*.

The mitochondrial free radical theory of ageing (MFRTA) is one of the most important theories of ageing; it proposes that ageing is caused by the destruction of macromolecules by mitochondrial reactive oxygen species (ROS) (Hekimi et al., 2011). Therefore, decreased ROS levels provide an important basis for assessing the abilities of organisms to delay ageing. The results showed that the green fluorescence of nematodes treated with *SSE* was substantially dimmer than for nematodes in the control group, which indicated that the ROS levels were noticeably reduced. This may be due to the increased SOD activity in nematodes due to the *SSE* treatments, while the polyphenolic compounds present in *SSE* may also play a role in ROS reduction.

**FIGURE 9:**
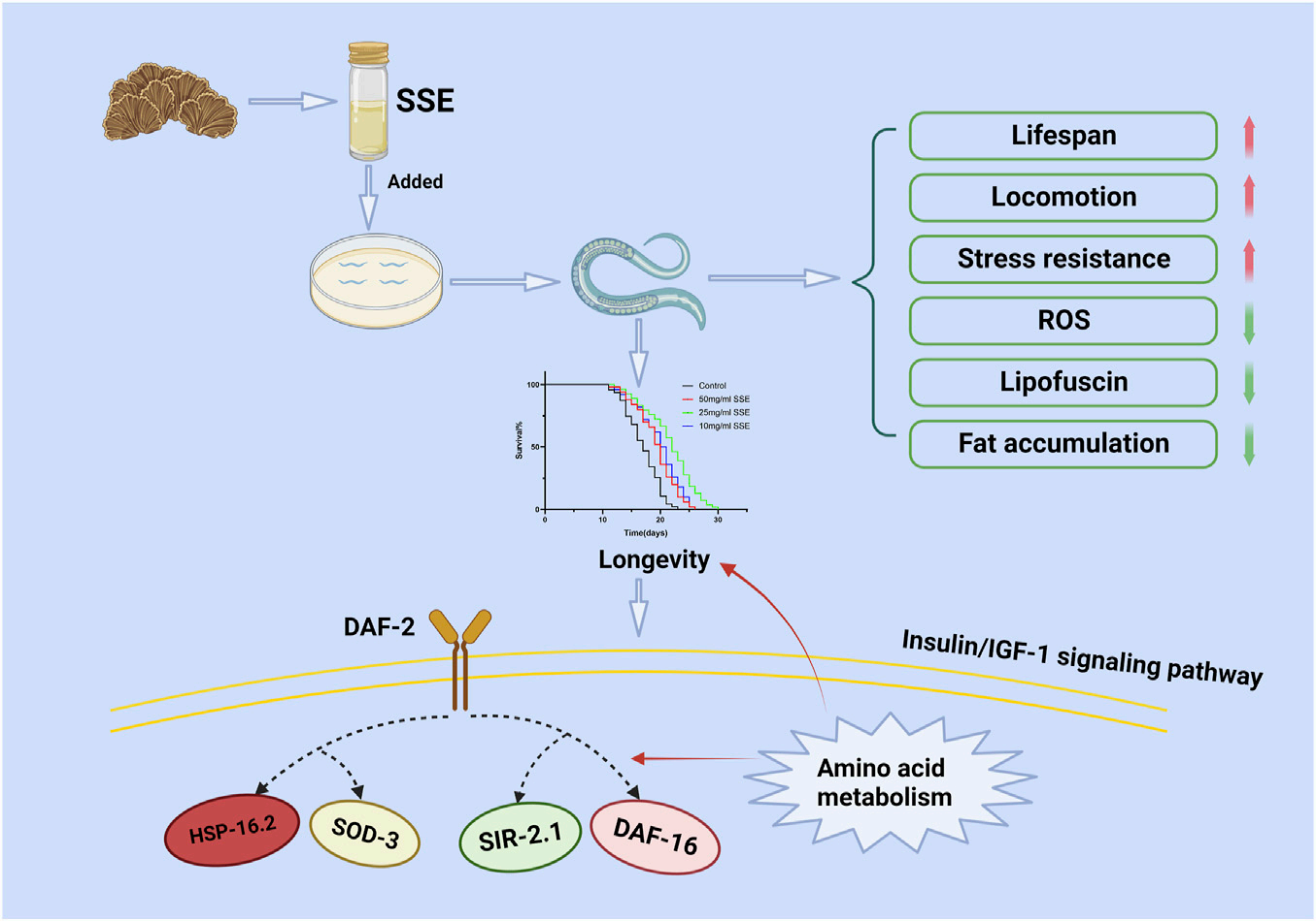
Possible molecular mechanism of the anti-ageing effect of *SSE*. Drawn using Biorender.

The real-time PCR results showed that *SSE* activated the expressions of *daf-16*, *daf-2* and *sir-2.1* in the IIS pathway. The insulin/insulin-like growth Factor 1 (IGF-1) signalling (IIS) pathway regulates lifespans in nematodes, mice and humans (Mukhopadhyay et al., 2006), and *daf-16* is the most important target of this pathway because it is activated by translocation of *daf-16* to the nucleus. *Daf-2* is the upstream gene of *daf-16* and regulates the expression of *daf-16* by a series of cascaded amplification reactions (Zecic and Braeckman, 2020). *Sir-2.1* is a cofactor of *daf-16* that can bind to *daf-16* to activate the expression of *daf-16*. We found that the *SSE* treatments upregulated the expression of *daf-16 via* the synergistic effects of *daf-2* and *sir-2.1*. Notably, the expression levels of sod-3 and *hsp-16.2*, the downstream genes of *daf-16*, were also upregulated to different degrees. These two genes are important for resistance to oxidation and heat shock, respectively (Figure 9). Meanwhile, the SSE-mediated lifespan extension was not observed in daf-16 and sir-2.1 mutant nematodes. These results suggest that daf-16 and sir-2.1 are the necessary condition for SSE extending the lifespan of *C. elegans*. Metabolomics results suggest that the regulation of lifespan by SSE also related to amino acid metabolism. Leucine activates the expression of *sir-2.1*, while other amino acids also rely on *daf-16* to extend lifespan. The change of amino acid metabolism is whether SSE enhances amino acid uptake capacity or activates amino acid generation pathway still needs to be verified in subsequent experiments.

## 5 Conclusion

We have shown that *SSE* treatments can substantially prolong the lifespans and improve the stress resistance of nematodes. Meanwhile, *SSE* can decrease the accumulations of age pigments, ROS and fat and strengthen the physiology of *C. elegans*, which when treated with *SSE,* showed increased expression levels of genes (e.g., daf-16, sir-2.1, daf-2, sod-3, and hsp-16.2), especially daf-16/sir-2.1, which can effectively resist ageing through the insulin/insulin-like growth Factor 1 (IGF-1) signalling (IIS) pathway. These results demonstrate that *SSE* has a beneficial effect on the lifespan of *C. elegans* and provides a theoretical basis for antiaging market applications of *S. Sanghuang*.

## Data Availability

The original contributions presented in the study are included in the article/Supplementary Material, further inquiries can be directed to the corresponding author.
