# Supplementary material for: Sanghuangporus sanghuang extract extended the lifespan and healthspan of Caenorhabditis elegans via DAF-16/SIR-2.1
Source: Front Pharmacol. 2023 Apr 20;14:1136897. doi: 10.3389/fphar.2023.1136897 (PMC10159060; doi:10.3389/fphar.2023.1136897)
Supplement: Supplementary file 1 [file Table1.DOCX]

Table S1 Survival time of different groups of nematodes

|  | **Mean Lifespan(d)** | **Max Lifespan (d)** |
| --- | --- | --- |
| Control 1 | 16.65 | 21 |
| Control 2 | 15.98 | 22 |
| Control 3 | 16.07 | 21 |
| 50 mg/mL SSE 1 | 18.15 | 24 |
| 50 mg/mL SSE 2 | 18.20 | 26 |
| 50 mg/mL SSE 3 | 18.35 | 24 |
| 25 mg/mL SSE 1 | 20.91 | 29 |
| 25 mg/mL SSE 2 | 20.39 | 28 |
| 25 mg/mL SSE 3 | 20.26 | 28 |
| 10 mg/mL SSE 1 | 18.91 | 26 |
| 10 mg/mL SSE 2 | 18.83 | 25 |
| 10 mg/mL SSE 3 | 18.82 | 24 |
